# A systematic review on anti-malarial drug discovery and antiplasmodial potential of green synthesis mediated metal nanoparticles: overview, challenges and future perspectives

**DOI:** 10.1186/s12936-019-2974-9

**Published:** 2019-10-03

**Authors:** Loick P. Kojom Foko, Francois Eya’ane Meva, Carole E. Eboumbou Moukoko, Agnes A. Ntoumba, Marie I. Ngaha Njila, Philippe Belle Ebanda Kedi, Lawrence Ayong, Leopold G. Lehman

**Affiliations:** 1 Department of Animal Biology, Faculty of Science, The University of Douala, P.O. Box 24157, Douala, Cameroon; 2 Department of Pharmaceutical Sciences, Faculty of Medicine and Pharmaceutical Sciences, The University of Douala, P.O. Box 2701, Douala, Cameroon; 3 Malaria Research Unit, Centre Pasteur Cameroon, P.O. Box 1274, Yaoundé, Cameroon; 4 Department of Biological Sciences, Faculty of Medicine and Pharmaceutical Sciences, The University of Douala, P.O. Box 2701, Douala, Cameroon

**Keywords:** Metal nanoparticles, Green synthesis, Antiplasmodial activity, Toxicity

## Abstract

**Background:**

The recent emergence in Southeast Asia of artemisinin resistance poses major threats to malaria control and elimination globally. Green nanotechnologies can constitute interesting tools for discovering anti-malarial medicines. This systematic review focused on the green synthesis of metal nanoparticles as potential source of new antiplasmodial drugs.

**Methods:**

Seven electronic database were used following the Preferred Reporting Items for Systematic Reviews and Meta-Analyses (PRISMA) guidelines.

**Results:**

A total of 17 papers were included in the systematic review. 82.4% of the studies used plant leaves to produce nanoparticles (NPs) while three studies used microorganisms, including bacteria and fungi. Silver was the main metal precursor for the synthesis of NPs. The majority of studies obtained nanoparticles spherical in shape, with sizes ranging between 4 and 65 nm, and reported no or little cytotoxic effect of the NPs. Results based on 50% inhibitory concentration (IC50) varied between studies but, in general, could be divided into three NP categories; (i) those more effective than positive controls, (ii) those more effective than corresponding plant extracts and, (iii) those less effective than the positive controls or plant extracts.

**Conclusions:**

This study highlights the high antiplasmodial potential of green-synthesized metal nanoparticles thereby underscoring the possibility to find and develop new anti-malarial drugs based on green synthesis approaches. However, the review also highlights the need for extensive in vitro and in vivo studies to confirm their safety in humans and the elucidation of the mechanism of action.

**Graphical abstract:**

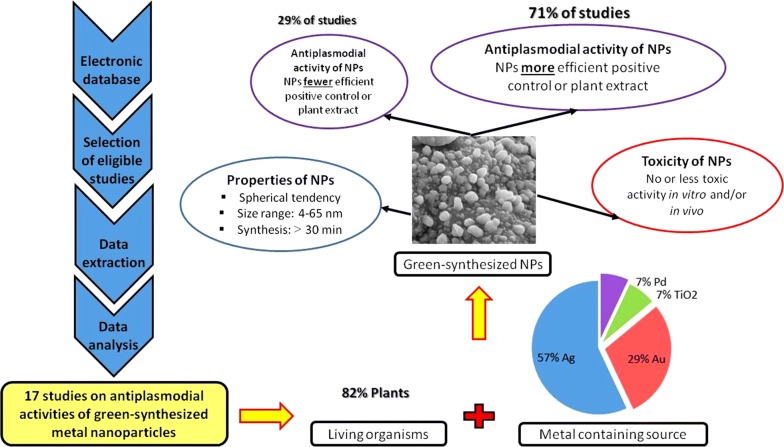

## Background

In 2015, nearly 9.2 million deaths occurred in Africa with 56.4% being due to communicable, maternal, perinatal or nutritional conditions and, malaria ranked as one of the most devastating infectious diseases characterized by intermittent high fevers, severe anaemia, convulsions, neurological complications such as brain injury and coma [1]. Malaria is caused by protozoan parasites of the genus *Plasmodium* that are transmitted to humans through the bite of infected female *Anopheles* mosquitoes [2]. *Plasmodium falciparum* causes most of the deaths, whereas *Plasmodium vivax* is the most widespread. *Plasmodium malariae*, *Plasmodium ovale*, *Plasmodium knowlesi*, and *Plasmodium cynomolgi* are other species that infect or cause disease in humans [2–4]. Malaria remains a very important public health problem, especially in sub-Saharan Africa where the disease has significantly delayed economic development. In 2017, approximately 219 million malaria cases and 435,000 related deaths were recorded worldwide; the majority (92%) of which occurred in sub-Saharan Africa [3].

Despite significant global efforts in the fight against malaria through increased funding for malaria research and development, delivery and scaling up of control interventions (diagnosis, prevention and treatment), the Global Technical Strategy (GTS) goals for malaria morbidity and mortality for 2020 are far from being achieved [3]. The World Malaria Report 2018 reported that only 70% of cases were avoided from 2000 to 2015, and also showed an increase in malaria cases in some countries from 2016 to 2017.

Unfortunately, one of the major barriers to successful global malaria control (GMC) is the emergence and the propagation of parasites resistant to currently used anti-malarial drugs. Artemisinin-based combination therapy (ACT), which is the most effective treatment available today, has been an integral part of the recent successes in GMC [2–4]. However, the future of these artemisinin-based combinations is endangered by the emergence of artemisinin resistant *P. falciparum* strains primarily reported in western Cambodia and subsequently in the Greater Mekong Subregion (GMS) and Southern China [5–7]. The circulation of artemisinin (ART) resistant parasites and/or resistant to partner drugs in ACT has greatly hindered the management of malarious patients and control strategies in these areas. Many studies reported increased failure rates following ACT due to the presence of ART-resistant parasites [8–10].

The resistance phenotype against artemisinin loci seems to be under positive selection within the propeller domain of the *P. falciparum kelch* (*k13*) gene, but other studies have indicated that additional single nucleotide substitutions on chromosomes 10, 13, and 14 may also be responsible for this resistance phenotype [11–13]. This suggest the exact genes which confer this delayed clearance or involved in artemisinin resistance are yet unknown, although 13 nonsynonymous mutations have been validated as associated markers [5, 14–19]. Moreover, mortality rates as well as recurrent malaria cases increased following the spread of artemisinin resistant parasites in these areas [1].

The emergence or spread of artemisinin resistance from Asia to Africa, as observed previously for older anti-malarial drugs including chloroquine and sulfadoxine–pyrimethamine [20–22], would be devastating to global malaria elimination efforts. Despite numerous fears on the potential emergence or spread of artemisinin resistance-associated *k13* mutations in Africa, the so far identified mutations are rare and unrelated to *k13* polymorphisms found to be associated with reduced susceptibility in Asia [5, 23–31]. Thus, in this situation and in the context where many anti-malarial treatments are paid for by non-profit organizations and governments, the future of malaria control and global elimination would depend on the ability of research and development to deliver the next generation of anti-malarial drugs [32]. If unsuccessful, this could greatly jeopardize the hope to efficiently control and eliminate malaria, particularly in the African continent as outlined in the Global Technical Strategy 2016–2030 [1].

Diverse strategies exist for the development of novel anti-malarial drugs, and some have come from living organisms. Basically the synthesis of metal NPs requests the combination of three elements namely: the metal source (generally noble metals such as silver, gold, palladium and titanium salt), the reducing agent and the capping agent. Metal nanoparticles are traditionally produced using chemical and physical methods. However, these methods are challenging as they are costly, time-consuming and request for utilization of reagents harmful to environment [33, 34]. In this regard, new NPs synthesis methods referred to as green synthesis have been developed to overcome these issues. Green synthesis consists in the production of metal NPs by exploiting the reducing and capping natural potential of biomolecules from living organisms such as plants and microorganisms. The method is simple, cost-effective and eco-friendly [33, 34].

Nanoproducts and metal nanoparticles are highly useful, safe in nature with numerous applications in renewable energies, catalysis, cosmetics, food, electronics, environmental remediation, biomedical devices and health [35, 36]. Metal NPs were mainly tested for their biocidal activity against bacteria [37–39], fungi [40, 41], and viruses [42, 43]. Little is reported on antiplasmodial potential of metal NPs [44]. In this systematic review, the living organisms mediated synthesis of nanoparticles (NPs) is presented as a source for new medicines to overcome the possible loss of ACT in the future. A recent systematic review by Barabadi and co-workers [45] addressed the utilization of biosynthesized NPs as control tool of malaria vectors and parasites. The authors did not address some of the gaps and challenges existing in this emerging line of research as well as the toxicity of green nanoparticles against non-target organisms (humans, for example). However, the authors reported interesting biocidal antiplasmodial activity of NPs but unfortunately, information concerning the efficacy of NPs compared to the positive control (anti-malarial drug or plant extract) is lacking.

Thus, data from 17 studies on the antiplasmodial activity of green-synthesized metal nanoparticles were comprehensively analysed with aims (1) to present commonly used biological material and main methodological aspects for green synthesis of metal nanoparticles; (2) to summarize the main findings of the selected studies; (3) to outline difficulties encountered in the synthesis of green-synthesized metal nanoparticles and, evaluation of their antiplasmodial and cytotoxic potential and, (4) to highlight future challenges and gaps in green technology driven anti-malarial drug discovery.

## Methods

### Data source and eligibility criteria

Two authors of the research team developed a strategy to search for articles to be included in the systematic review. Between 25th September and 25th November 2018 seven electronic databases including Medline, Scopus, Excerpta Medica Database (EMBASE), African Index Medicus, Popline, Africa wide information and the Cochrane library were used to search for potentially eligible publications. Supplemental sources included Boolean operators that helped to conduct more efficient searches from these databases. In addition, search engines such as Google and Google Scholar were also used to identify all potentially eligible publications. Another search was performed between 7th February and 30th March 2019.

The review included (i) studies focused on the effectiveness of green-synthesized metal-based nanoparticles against malaria parasites, (ii) published peer reviewed and research articles between January 1999 and March 2019 and, written in English or French. Articles were thereafter independently reviewed, rated and data were abstracted by two persons. Studies focused mainly on malaria vectors, letters to the editor, editorials, conference papers, preprints, reports or comment publication type sources were excluded from the review.

### Screening strategy

Titles and abstracts of retrieved studies from electronic database of interest were reviewed by two persons and the terms used during the search process were distributed in four groups (Table 1) and combined with Boolean operators “AND”/“OR” during the searching using the above mentioned electronic databases. The full texts of each study were retrieved, analysed and the screened studies were included in the review. Corresponding authors of relevant documents were asked to provide full texts when not free or inaccessible. When it was not possible, i.e. non-reply or negative reply from corresponding authors these full texts were purchased. In addition, the references list of relevant documents was also examined to increase the chances of finding eligible papers. The Preferred Reporting Items for Systematic Reviews and Meta-Analyses (PRISMA) flowchart was used to depict the entire stepwise process of screening strategy (Additional files 1 and 2) [46].

**Table 1:** Word groups used to screen relevant documents in the different electronic databases used

Nanoparticles-producing living organisms	Diseases or parasites	Word groups	Inputs and outcomes
		Nature and type of synthesis of nanoparticles	
‘Plant^a^’ OR ‘Bacteria^a^’ OR ‘Mushroom’ OR ‘Fungi^a^’ OR ‘Worms’ OR ‘Helminths^a^’ OR ‘Microorganism’ OR ‘Microbial^a^’	‘Malaria^a^’ OR ‘Plasmodium^a^’ OR ‘Plasmodium falciparum^a^’ OR ‘Plasmodium berghei^a^’	‘Green synthesis^a^’ OR ‘Biological synthesis^a^’ OR ‘Biosynthesis’ OR ‘Plant-mediated’ OR ‘Metal-based’ OR ‘Nanoparticles^a^’ OR ‘Metal nanoparticles^a^’ OR ‘Biometallic nanoparticles^a^’ OR ‘Gold nanoparticles’ OR ‘Silver nanoparticles’ OR ‘Copper nanoparticles’ OR ‘Platinum nanoparticles’ OR ‘Titanium’ OR ‘Palladium’	‘In vitro activity’ OR ‘In vitro potential’ OR ‘In vivo activity’ OR ‘In vivo potential’ OR ‘antimalarial activity’ OR ‘Antimalaria^a^’ OR ‘Antiplasmodial^a^ activity’ OR ‘Effectiveness’ OR ‘Assessment’ OR ‘Effect’ OR ‘Properties’

^a^Medical Subject Headings (MeSH) terms used to make search in PubMed

### Data of interest

All information collected in the different articles collected from the seven different databases used are classified into six major groups (Table 2). Two investigators independently extracted data and any discrepancies were resolved through discussion and consensus.

**Table 2:** Data of interest extracted from the included studies

Information groups	Data retrieved
Group 1: General information	Name of the first author
	The year of publication
	Characteristics of living organism used for synthesis of metal NPs (scientific name, common name, family, morphological type, type of microorganism and origin of isolation
Group 2: Data on mode of synthesis of NPs	Biological material
	Quantity of biological material
	Metal source
	Extraction solvent
Group 3: Methods used for physical characterization of metal NPs	Shape
	Size
	Size distribution
	Silver content
	Stability
	Structure-crystallinity
Group 4: Data on method used for evaluation antiplasmodial activity of NPs	Study design (in vivo, in vitro)
	Nature and origin of *Plasmodium* strain
	Negative and positive controls
	NPs doses tested
Group 5: Data on physical properties of NPs	Size, shape, color
	Maximum absorption peak
	Aggregation phenomenon
Group 6: Data on antiplasmodial efficacy of NPs and their cytotoxic activity	50% inhibitory concentration (IC_50_) and/or percentage parasite growth inhibition
	Tests used to appraise the NPs toxicity
	Side effects recorded

### Data verification for consistency

Data of interest were independently keyed in an Excel spreadsheet (Microsoft Office 2016, USA) by two persons to ensure internal quality control of database. These data were also checked for consistency by two additional persons for external quality control of database. When discrepancy between the two Excel sheets occurred, two more people checked the data again.

## Results

### Characteristics of the studies included in the review

**Fig. 1:**
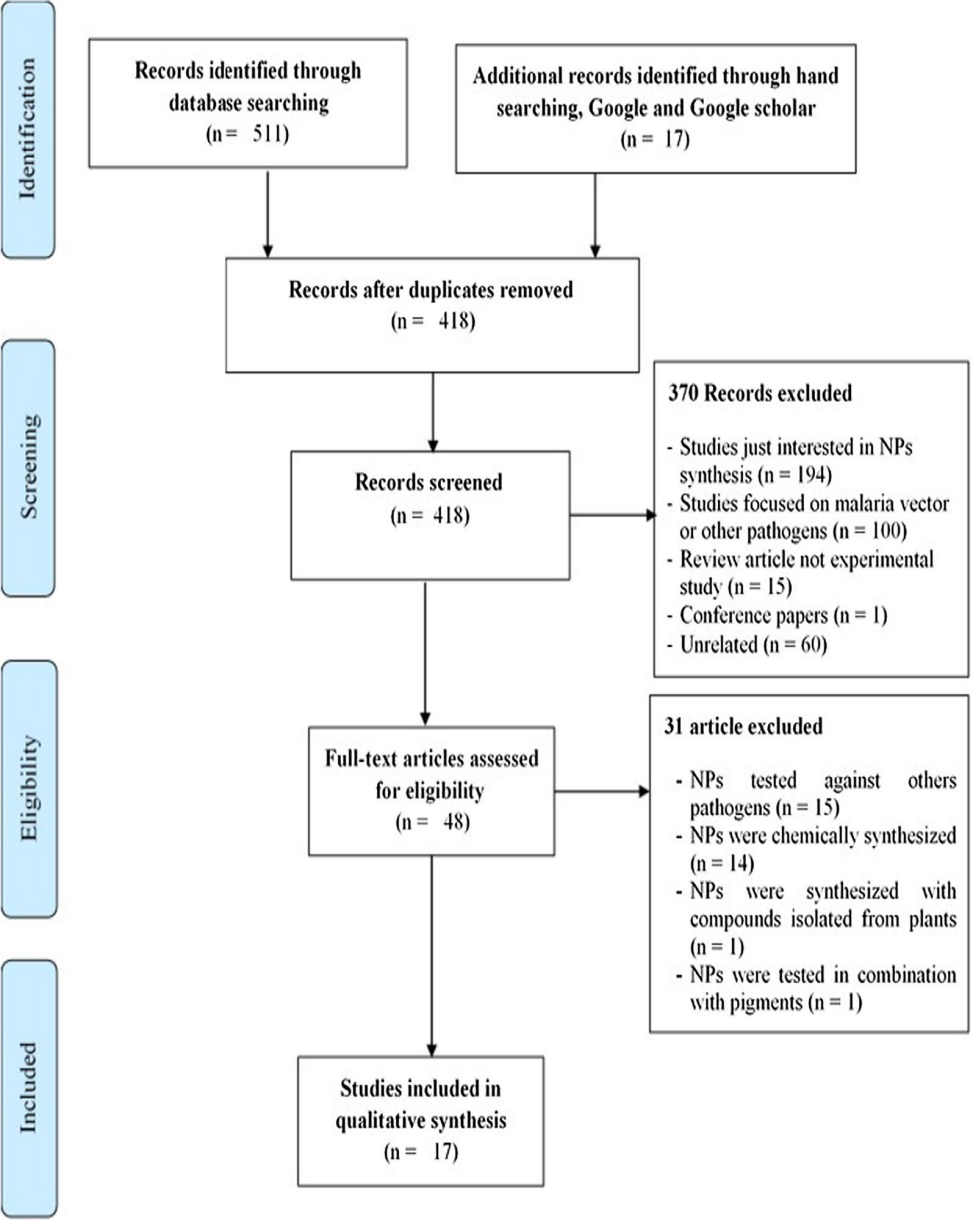
PRISMA flowchart on the selection strategy of eligible articles for the review

In total, 17 studies were eligible based on the selection process summarized in Fig. 1 [47–63]. All these studies clearly synthesized, characterized and assessed the antiplasmodial potential of biologically produced metal-based nanoparticles (NPs). Fourteen of them used plants as biological material for producing NPs, while the three remaining used microorganism especially bacteria (Table 3). Most of included studies (16/17; 94.11%) were conducted by Indian research teams.

**Table 3:** Characteristics of plants and microorganisms used for synthesis of metal nanoparticles

Authors [Reference]	Country	Plants used for synthesis of NPs (Family)	Common/International name	Morphology
Panneerselvam et al. [47]	India	*Andrographis paniculata* (Burm.f.) Nees (Acanthaceae)	King of Bitters	Herbaceous plant
Ponarulselvam et al. [48]	India	*Catharanthus roseus* Linn. G. Don (Apocynaceae)	Cayenne jasmine, Old maid	Herbaceous plant
Mishra et al. [49]	India	Ashoka (*Sarca indica*) (Caesalpiniaceae) Neem (*Azadirachta indica* A. Juss)(Meliaceae)	Ashoka, Neem	Tree
Panneerselvam et al. [50]	India	*Euphorbia hirta* (Euphorbiaceae)	Pill-bearing/hairy/garden spurge, asthma plant	Herb
Rajakumar et al. [51]	India	*Eclipta prostrata* L. (Asteraceae)	False daisy	Herbaceous plant
Murugan et al. [52]	India	*Ulva lactuca* (Chlorophyceae)	Sea lettuce	Algae
Subramaniam et al. [53]	India	*Couroupita guianensis* (Lecythidaceae)	Cannonball tree	Tree
Panneerselvam et al. [54]	India	*Pteridium aquilinum* L. Kuhn (Dennstaedtiaceae)	Bracken fern	Herb
Murugan et al. [55]	India	*Codium tomentosum* (Codiaceae)	Velvet horn, Spongeweed	Herb
Murugan et al. [56]	India	*Azadirachta indica* A. Juss (Meliaceae)	Village pharmacy, Neem	Tree
Dutta et al. [57]	India	*Syzygium jambos* (L.) Alston (Myrtaceae)	Rose apple	Tree
Sardana et al. [58]	India	Tulsi (*Ocinum sanctum*) (Lamiaceae) Neem (*Azadirachta indica* A. Juss) (Meliaceae)	Tulsi, Neem	Herb tree
Gandhi et al. [59]	India	*Momordica charantia* (Cucurbitaceae)	Bitter melon, bitter apple	Herbaceous plant
Rotimi et al. [60]	South Africa	*Callistemon citrinus* (Myrtaceae)	Lemon Bottlebrush	Herb

Authors [Reference]	Country	Microorganisms used for synthesis of NPs	Nature of the microorganism	Site of isolation
Kharthik et al. [61]	India	*Streptomyces* sp LK-3 (JF710608)	Bacterium (*Actinobacterium*)	Marine sediments
Jaganathan et al. [62]	India	*Eudrilus eugeniae*	Earthworm	Not specified
Murugan et al. [63]	India	*Magnetospirillum gryphiswaldense* MSR-1 (DSM6361)	Magnetotactic bacterium	Laboratory strain

### Information on living organism used for synthesis of metal nanoparticles

Globally, 17 plant species distributed into 16 families were investigated for their ability to elicit nano-sized materials with antiplasmodial properties (Table 3). These plants included *Andrographis paniculata*, *Azadirachta indica* and *Pteridium aquilinum*. These are popularly known as “King of Bitters”, “Neem” and “Bracken fern” respectively by populations who use them. The morphological type of plants included tree, herb, herbaceous herb and algae (Table 3). Of the three studies that used microorganisms, two focused on bacteria and the remaining one on worms. Bacteria consisted of *Streptomyces* sp LK-3 (JF710608) and *Magnetospirillum gryphiswaldense* which were isolated from marine sediments and laboratory-maintained respectively (Table 3).

### Methods used for green synthesis of metal nanoparticles within included studies

Leaves were used as biological material for synthesis of metal NPs in 11 out of 14 studies having used plants. The other plant parts included flowers, seeds and barks [50, 53, 57]. Decantation was predominantly used by authors to produce aqueous extracts which had to be mixed with metal precursor for NPs synthesis (Table 4). Indeed, biological material (4–10 g) was boiled following careful washing (tap water and double-distilled water) and cutting. Water was mainly used as extraction solvent and the mixture was then decanted and filtered using Whatman N°1 filter paper (Table 4). The mode of preparation of NPs among studies having used microorganisms was available in two studies. For instance, Kharthik and colleagues inoculated their bacterium of interest, incubated with metal precursor in aqueous medium consisting of 50% sea water and the mixture was then centrifuged [61].

**Table 4:** Synthesis, characterization methods, characteristics and antiplasmodial activity assessment of metal nanoparticles

Items	Parameters	Categories	References
Mode of preparation	Part used for NPs synthesis	Leaves	[47–50, 52, 53, 57–60]
		Flowers	[53]
		Seeds	[55, 56, 60]
		Barks	[57]
		Whole organism	[61–63]
	Extraction solvent	Water	[47, 48, 50–60]
		Tris–HCl buffer	[49]
	Main method of preparation	Washing, cutting, boiling, decantation, filtration	[47–56, 59]
Physico-chemical characterization of NPs	Plasmon resonance	UV–Vis	[47, 48, 50–63]
	Shape, size and size distribution	FESEM/SEM, EDX, EDAX	[47, 48, 50–56, 60–63]
		HRTEM/TEM, SAED	[49, 51, 57–61, 63]
	Silver content	AAS	[49]
	Interface NPs-metabolites	FTIR	[49, 51–53, 55–57, 59, 60, 62, 63]
	Size distribution	DLS	[49, 59]
	Structure-crystallinity	XRD	[47–57, 61]
	Stability	Zeta potential	[47, 51, 53, 55]
Characteristics of NPs	Metal source	AgNO_3_	[47–50, 52, 54–58, 61, 63]
		HAuCl_4_	[53, 57, 60, 61]
		Pd(OAc)_2_	[51]
		TiCl_4_	[59]
	Time of production	< 30 min	[50, 58, 59, 61]
		> 30 min	[47, 49, 51, 53–56]
	Shape	Spherical or mainly spherical	[47–51, 54, 56–59, 62]
		Other shapes (cubical, polygonal, triangular, oval, ellipsoidal, rectangular)	[50–53, 55, 57–59, 61]
	NPs aggregation phenomenon	Yes	[49, 56]

*AAS* atomic absorption spectroscopy, *DLS* dynamic light scattering, *DRIFT* diffuse reflectance infrared Fourier transform, *EDS* Electron Diffraction Spectrophotometer, *FESEM* field emission scanning electron microscopy, *FTIR* Fourier-transform infrared, *HR-TEM* high resolution transmission electron microscopy, *SAED* size and elected area diffraction, *SEM* scanning electron microscopy, *TEM* transmission electron microscopy, *EDAX* energy dispersive X-ray, *HF* health facility

### Methods used for studying nanoparticles

The physical characterization of metal nanoparticles was studied on four aspects namely shape, size and distribution size, chemical composition, and structure and stability (Table 4). Ultraviolet spectroscopy (UV–Vis) proved the formation of the nanoparticles by showing the characteristic plasmon vibration. Scanning electron microscopy (SEM) and transmission electron microscopy (TEM) were used to determine the shape and size of the nanoparticles. SEM is coupled with energy dispersive X-ray spectroscopy (EDX, EDS) which provides elemental mapping in terms of atomic composition while TEM is coupled with selected area electron diffraction (SAED) which shows crystallographic planes. Fourier-transform infrared spectroscopy (FTIR) was used to study the nanoparticles/secondary metabolites interface by providing molecular vibrations. X-ray diffraction (XRD) was used for nature, crystallinity as well as shape and size determination. Dynamic light scattering (DLS) provides size distributions in term of hydrodynamic radius. Few studies studied the nanoparticles stability by determining their Zeta potential and the silver content by Atomic absorption spectroscopy (AAS) [53, 57, 59, 61].

### Characteristics of plant-synthesized nanoparticles

As depicted in Table 4, silver nitrate (AgNO_3_) was mainly used as metal precursor for synthesis of plant-related nanoparticles. Other noble metals including gold, titanium and palladium were also used for NPs synthesis [51, 53, 57, 59, 61]. The colour change outlining the obtainment of NPs was achieved between 10 and 150 min after mixture between precursor metal and plant extract.

UV–Vis spectroscopy appears as one of the key method to investigate nanoparticles behaviour such as formation, development or aggregation. Characteristic plasmon vibration occurs because of the free oscillation of electrons at the metallic surface. They are situated at 400–450 nm for silver [47–50, 52, 54–58], 540 and 560 nm for gold [51, 61], 360 for TiO_2_ [59].

A majority of studies obtained spherical-shaped nanoparticles (Table 4). Globally, the size of NPs ranged between 4 and 65 nm and a few studies reported an aggregation phenomenon during synthesis [49, 56]. Some studies reported the appearance of additional Braggs peaks [48–50, 56]. The presence of energy dispersive X ray-related signals associated with oxygen or carbon atoms [52, 56], while additional chlorine signal may appear too [54]. Selected area electron diffraction (SAED) showed diffraction dots and crystallographic planes of the obtained nanoparticles (Table 5) [57].

**Table 5:** FTIR shows characteristic vibrations and translations

Authors [Reference]	Main IR characteristics							Particular characteristics	
	O–H stretch N–H stretch	C–H stretch	CN stretch	C=C stretch N–H bend C=O stretch	N–O stretch	C–H bend C–N stretch C–O stretch N–H bend	C–O stretch	C–O stretch C–N stretch	
Rajakumar et al. [51]	3361				1540	1399		1049	
Mishra et al. [49]	3622			1699			1388	1043	
Murugan et al. [52]	3280		2359					1092–1027	
Jaganathan et al. [62]^a^	3455	2920		1639	1555	1407			
Subramaniam et al. [53]	3421		2362	1641	1514	1456			
Murugan et al. [55]	3416; 3402			1640/1635					
Murugan et al. [56]	3479; 3402		2359					1092–1027	
Dutta et al. [57]	3341–3308	~ 2800				~ 1400			
Murugan et al. [63]	3273	2921/2924	2355	1728					
Gandhi et al. [59]	3377	2941		1695		1417	1293	1078	700–450 Ti–O–Ti
Rotimi et al. [60]	3400			1680					500 Au–O

The table shows only selected bands discussed by the authors, ^a^earthworms

Powder X-ray diffraction is one of the most important characterization tool used in solid state chemistry [64]. It is used to determine the nature of the crystalline phases and then of the synthesized nanoparticles starting from the biological extracts (plant, earthworm). More antiplasmodial properties have been evaluated with nanosilver (57%) and nanogold (29%) (Table 4). This determination is possible by comparison of the obtained pattern to the International Centre for Diffraction Data (ICDD) patterns, formerly the Joint Committee on Powder Diffraction Standards (JCPDS) patterns. If the biosynthesis leads to pure palladium, gold or titanium dioxide nanoparticles, it is not the case for silver where nanosilver, silver chloride nanocrystallites or their mixture are obtained [35, 36, 43].

FTIR was carried out to investigate biomolecules extracts at the metallic interface of silver, gold, palladium and the metal oxide interface for titanium. The method shows molecular vibrations at the surface or the synthesized nanoparticles. FTIR spectroscopy revealed absorption frequencies that can be well correlated with characteristic tables. For example, O–H (stretch, H-bonded) at 3200–3600 and C–O (stretch) at 1050–1150 for alcohols (Table 5). C–H (stretch) at 2860–3000 and –C–H (bending) at 1350–1480 for alkanes. C=C (stretch) ae 1620–1680 for alkenes. N–H (stretch) at 3300–3500 and N–H (bending) at 1600 but also C–N (stretch) at 1080–1360 for amines. C–H (stretch) at 3000–3100 and C=C (stretch) at 1400–1600 for aromatics or C=O (stretch) at 1670–1820 for carbonyls [65].

### Evaluation methods and findings on antiplasmodial activity of nanoparticles

The studies were mainly designed as in vitro even though a few studies were in vivo [51, 59], or a combination of both [56, 63]. Most studies evaluated the susceptibility of laboratory strains of *P. falciparum,* such as INDO (CQ-resistance), 3D7 (CQ-sensitive), FcB1/Colombia (CQ-sensitive) and Dd2 (CQ-sensitive) using chloroquine as positive control (Table 3). Negative controls were included in the study design and consisted of distilled water, uninfected and infected red blood cells or medium culture. A few studies collected *P. falciparum* field isolates from patients attending health facilities [54, 55, 57]. *Plasmodium berghei* was used in studies based on animal model for appraising the malarial susceptibility and nanoparticles were administered either orally [56], or intraperitoneally [61].

The percentage of parasite growth suppression and 50% inhibitory concentration (IC_50_) were used as endpoints for evaluation of antiplasmodial activity of nanoparticles (Table 6). Panneerselvam and co-workers reported a reduction in parasite growth rate by 26% to 83% at doses 25 µg/mL and 100 µg/mL respectively [50]. A lower antiplasmodial activity comprises between 6.4 and 42.8% was reported by Murugan and colleagues [56]. Results based on IC_50_ were very contrasted between studies but generally, these met into three categories namely (i) nanoparticles were more efficient than positive control [52–56], (ii) nanoparticles were more efficient than plant extract [49, 51, 56, 57], and (iii) nanoparticles were less efficient than positive control (chloroquine) or plant extract [53–55, 57]. Nine of eleven studies having used chloroquine as control found that metal NPs were more efficient [49, 51–56, 62, 63]. For instance, Jaganathan et al. found their nanoparticles had IC_50_ of 49.3 µg/mL and 55.5 µg/mL against *P. falciparum* 3D7 (chloroquine-sensitive) and INDO (chloroquine-resistant) strains respectively compared to chloroquine (81.5 µg/mL and 86.5 µg/mL respectively) [62]. Murugan et al. reported an IC_50_ of 63.18 µg/mL and 69.24 µg/mL for nanoparticles compared to 82.41 µg/mL and 86.12 µg/mL for extracts against 3D7 and INDO strains respectively, thus outlining a higher antiplasmodial activity of nanoparticles compared to plant extract (Table 6) [56].

**Table 6:** Antiplasmodial effectiveness of synthesized nanoparticles

Authors [Reference]	Results on effectiveness of NPs	Results on used controls
Panneerselvam et al. [47]	Parasite growth inhibition rate ranged from 26% (25 µg/mL) to 83% (100 µg/mL) IC_50_ = 50 µg/mL	Not computable
Ponarulselvam et al. [48]	Parasite growth inhibition rate ranged from 20% (25 µg/mL) to 75% (100 µg/mL) IC_50_ = 63.64 µg/mL^a^	Not computable
Kharthik et al. [61]	Nearly 40% inhibition was observed at dose 8 mg/kg/bw	Not computable
Mishra et al. [49]	IC_50_ = 8 µg/mL (Ashoka)	IC_50_ = 0.5 µg/mL (AgNO_3_)
	IC_50_ = 30 µg/mL (Neem)	No activity up to 40 µg/mL (extract)
Panneerselvam et al. [50]	Parasite growth inhibition rate ranged from 26.2% (20 µg/mL) to 100% (100 µg/mL) IC_50_ = 51.46 µg/mL^a^	Not computable
Rajakumar et al. [51]	IC_20_ = 4.34 µg/mL, IC_50_ = 8.704 µg/mL, IC_90_ = 18.49 µg/mL	AE: IC_20_ = 1.90, IC_50_ = 10.29 and IC_90_ = 64.11 µg/mL
		Pd(AcO)_2_: IC_20_ = 4.49, IC_50_ = 9.84 and IC_90_ = 23.046 µg/mL
Murugan et al. [52]	IC_50_ = 76.33 µg/mL (3D7), IC_50_ = 79.13 µg/mL (INDO)	CQ: IC_50_ = 80 µg/mL (3D7), IC_50_ = 85 µg/mL (INDO)
Jaganathan et al. [62]	IC_50_ = 49.3 µg/mL (3D7), IC_50_ = 55.5 µg/mL (INDO)	CQ: IC_50_ = 81.5 µg/mL (3D7), IC_50_ = 86.5 µg/mL (INDO)
Subramaniam et al. [53]	IC_50_ = 69.47 µg/mL (3D7), IC_50_ = 76.33 µg/mL (INDO)	CQ: IC_50_ = 80 µg/mL (3D7), IC_50_ = 90 µg/mL (INDO)
		AE: IC_50_ = 43.21 µg/mL (3D7), IC_50_ = 51.16 µg/mL (INDO)
Panneerselvam et al. [54]	IC_50_ = 78.12 µg/mL (3D7), IC_50_ = 88.34 µg/mL (INDO)	CQ: IC_50_ = 85 µg/mL (3D7), IC_50_ = 90 µg/mL (INDO)
		AE: IC_50_ = 62.04 µg/mL (3D7), IC_50_ = 71.16 µg/mL (INDO)
Murugan et al. [55]	IC_50_ = 72.45 µg/mL (3D7), IC_50_ = 76.08 µg/mL (INDO)	CQ: IC_50_ = 80 µg/mL (3D7), IC_50_ = 85 µg/mL (INDO)
		AE: IC_50_ = 51.34 µg/mL (3D7), IC_50_ = 65.17 µg/mL (INDO)
Murugan et al. [56]	Parasite growth inhibition rate ranged from 6.4 to 42.8% IC_50_ = 63.18 µg/mL (3D7), IC_50_ = 69.24 µg/mL (INDO)	CQ: Parasite growth reduced from 51.2% up to 53.6%
		IC_50_ = 90 µg/mL (3D7), IC_50_ = 98.5 µg/mL (INDO)
		AE: Parasite growth reduced from 15.2% up to 58.6%
		IC_50_ = 82.41 µg/mL (3D7), IC_50_ = 86.12 µg/mL (INDO)
Dutta et al. [57]	AgNP: IC_50_ = 24.22 ± 2.44 µg/mL (Bark, 3D7) IC_50_ = 29.09 ± 2.54 µg/mL (Bark, Dd2) IC_50_ = 28.97 ± 3.21 µg/mL (Leaf, 3D7) IC_50_ = 34.49 ± 1.42 µg/mL (Leaf, Dd2)	AE: IC_50_ = 43.49 ± 3.23 µg/mL (Bark, 3D7) IC_50_ = 47.66 ± 3.97 µg/mL (Bark, Dd2)
		IC_50_ = 51.70 ± 1.29 µg/mL (Leaf, 3D7) IC_50_ = 53.37 ± 2.86 µg/mL (Leaf, Dd2)
	AuNP: IC_50_ = 49.54 ± 2.34 µg/mL (Bark, 3D7) IC_50_ = 51.63 ± 2.55 µg/mL (Bark, Dd2) IC_50_ = 45.49 ± 1.40 µg/mL (Leaf, 3D7) IC_50_ = 49.38 ± 3.04 µg/mL (Leaf, Dd2)	CQ: IC_50_ = 0.371 µg/mL (3D7), IC_50_ = 1.8 µg/mL (Dd2)
Murugan et al. [63]	IC_50_ = 83.32 µg/mL (3D7), IC_50_ = 87.47 µg/mL (INDO)	CQ: IC_50_ = 92 µg/mL (3D7), IC_50_ = 96 µg/mL (INDO)
Sardana et al. [58]	IC_50_ = 0.313 to 1.692 µM (3D7)	Not applicable
Gandhi et al. [59]	IC_50_ = 53.42 µg/mL (3D7), IC_50_ = 59.71 µg/mL (INDO)	CQ: IC_50_ = 0.021 µg/mL (3D7), IC_50_ = 0.258 µg/mL (INDO)
Rotimi et al. [60]	NPs were inactive against malaria parasites (% viability < 20%)	Not specified

*NPs* nanoparticles, *AgNPs* silver nanoparticles, *AuNPs* gold nanoparticles, *IC*_*20*_ 20% inhibitory concentration, *IC*_*50*_ 50% inhibitory concentration, *IC*_*90*_ 90% inhibitory concentration, *CQ* chloroquine, *AE* aqueous extract

^a^IC_50_ was calculated using data coming from the study

### Cytotoxicity of nanoparticles

As presented in Table 7, seven out seventeen studies included cytotoxicity analysis of synthesized nanoparticles [49, 51, 61]. Of the seven studies, four reported no or little deleterious effect of nanoparticles on used cell lines [49, 50, 57, 59]. Conversely, the remaining studies three reported important adverse effects including tissue damages, behavioural changes, changes in physical appearance, deaths of laboratory animals [61], necrosis and cytopathic effects [51], and apoptosis [62] (Table 7).

**Table 7:** Evaluation of toxicity of nanoparticles synthesized

Authors [Reference]	Used methods	Results
Kharthik et al. [61]	Brine shrimp lethality assay	No toxicity up to 8 mg/kg/bw
	Histological analysis	Tissue damages were reported at doses 12, 16 and 20 mg/kg/bw
	Search for any signs of toxicity	Deaths, behavioural changes, changes in physical appearance observed at doses 8, 12, 16 and 20 mg/kg/bw
Mishra et al. [49]	Haemolysis assay	No signs of haemolysis up to 40 µg/mL (MHC_10_ > 40 µg/mL)
Rajakumar et al. [51]	MTT assay using Hep-G2 cell line	Cellular toxicity (necrosis and cytopathic effects) of 8.5%, 24%, 48%, 65% and 76.5% at doses 1, 10, 100, 250 and 500 µg/mL respectively (more toxic than Pd (OAc)_2_ and plant extract)
Jaganathan et al. [62]	MTT assay using Hep-G2 cell line	Viability of Hep-G2 cells decreased when tested doses of NPs increased (IC_50_ = 25.96 µg/mL)
	Apoptosis assay	NPs induced apoptosis which increased significantly from 1.6 to 7.8% at doses 1.88 µg/mL and 30 µg/mL respectively
Dutta et al. [57]	MTT assay using HeLa and L6 lines	Insignificant toxicity against the both cell lines (IC_50_ > 200 µg/mL and > 250 µg/mL)
Gandhi et al. [59]	Non-target organism assay	NPs did not exhibit any noticeable toxicity on *Poecilia reticulata* after 24 h of exposure
Rotimi et al. [60]	Keusch et al. assay using HeLa lines	NPs were not toxic (% cell viability 89.66% ± 1.55%)

*NPs* nanoparticles, *IC*_*50*_ 50% inhibitory concentration, *bw* body weight, *MHC*_*10*_ minimum haemolytic concentration resulting in 10% haemolysis, *PBMCs* peripheral mononuclear cells, *MTT* 3-(4,5-dimethylthiazol-2-yl)-2,5-diphenyltetrazolium bromide

## Discussion

This systematic review focused on studies having evaluated the antiplasmodial activity of biologically synthesized metal nanoparticles. The production of nanoparticles (NPs) using living beings also known as green synthesis is much more interesting as it deals with environmental and economic issues. Indeed, this approach is environment friendly, rapid, nontoxic in most cases, cost-effective and easily scaled up for large scale production of NPs compared to their chemical and physical counterparts [35, 66]. In addition, compounds used for NPs chemical synthesis such as sodium borohydride (NaBH_4_) or Tollen’s reagent are non-biodegradable and very harmful to humans and expose them to cancer for instance [67].

Green synthesis of metal nanoparticles was mainly done using plant extracts. The utilization of plants is more advantageous as it limits the risk of biohazard and reduces costs imposed by isolation, purification of microorganisms as well as maintaining cell cultures [35, 68]. Furthermore, the critical need for creation of highly aseptic conditions and their maintenance impedes the possibility of using microbe-synthesized nanoparticles in a large-scale production perspective [35, 68]. Benelli [69] concluded in a precedent review that carbonyl groups had the stronger ability to bind metals, indicating that the proteins could form a capping layer on AgNPs, preventing agglomeration and thereby stabilizing the medium. Other molecules like (poly)phenols of enzymes and polysaccharides or flavonoids of proteins could perform that same role and build the metal interfaces.

Silver was mainly used as metal precursor for the synthesis of nanoparticles. This can be due to interesting properties of this atom such as its wide antimicrobial activity and chemical stability [70]. The synthesis leads generally to pure silver, silver chloride nanocrystallites or a mixture of both. The biosegregation of those entities by plant extract is not described in literature [71]. This atom has been known for having biocidal action against a broad range of microorganisms in ancient times. Nowadays, silver ions are used in a large number of medical situations including catheter disinfection, water purification, food hygiene and dental work for control of bacterial growth [72, 73].

The colour change outlining the obtainment of NPs was achieved between 10 and 150 min after mixture between precursor metal and plant extract. This colour change is attributed to the surface plasmon resonance phenomenon which occurs when free electrons present on the surface of nanoparticles enter in resonance with the wavelength of the incident light [57].

Nanoparticles were mainly spherical in shape even though other shapes were also reported. Furthermore, their size distribution was large enough. Both physical parameters are responsible for distinctive physico-chemical properties of NPs which underlie their biological activities against microorganisms [74–76]. The formation of NPs involves two stages namely (i) nucleation where nuclei form by self-assemblage of atoms and (ii) subsequent growth of this nuclei into a nanosized particle. Tran et al. [74] demonstrated that the size and shape of Ag-NPs were strongly dependent on these stages. Indeed, it is more likely to have monodispersed nanoparticles with uniform size distribution if all nuclei form at the same time. As a result, these nuclei will need to have the sale subsequent growth [74]. Additionally, factors such as reaction parameters (pH, ionic force, osmotic pressure and temperature), the nature of stabilizing agent and surface plasmon resonance can influence the shape and size of nanoparticles [77–80].

Importantly, a few studies reported an aggregation phenomenon during synthesis of metal nanoparticles outlining that nanoparticles were not stable during and/or after synthesis. These studies did not include methods such as energy dispersive X-ray (EDX) in their design in order to predict any possibility of aggregation. This phenomenon modulates the particular physico-chemical properties of nanosized particles and accordingly their biological actions [81]. However, a few authors outlined the importance of this phenomenon in toxicity against pathogenic microorganisms such as *Escherichia coli* induced by gold-based nanoparticles upon their intracellular penetration [82].

The studies were designed as in vitro, in vivo or a combination of both. In vitro studies have advantages to appraise the intrinsic susceptibility of malaria parasite to drugs compared to in vivo studies which results are strongly dependent on level of anti-malarial immunity of host. If not taken into account, one can believe illusively the effectiveness of tested molecules especially nanoparticles. Besides, most in vitro designed studies appraised the susceptibility of laboratory strains of *P. falciparum* such as INDO, 3D7 and Dd2. In a context of multidrug resistance in malaria parasites, it would be more interesting to test metal NPs against of *P. falciparum* field isolates in order to objectively appreciate their antiplasmodial action [47, 48, 50]. In the context of emergence and spread of resistance of malaria parasites to artemisinin and its derivatives, the possibility to develop new medicines through methods such as green nanotechnology is of utmost importance and interest. Furthermore, *P. falciparum* laboratory strains resistant to ART and its derivatives could be used as control instead of the above mentioned laboratory strains.

Most studies included in the review found that synthesized nanoparticles had antiplasmodial potential higher than used controls (chloroquine, extract). This finding indicates that these nanomaterials can be valuable tools for discovering and designing new medicines. The antiplasmodial activity of nanoparticles can be attributed to the presence of biological compounds such as flavonoids, alkaloids, terpenes, lignans, terpenoids, steroids, coumarins, phenolic acids, xanthones, proteins and anthraquinones [48, 62]. The Fourier-transform infrared spectroscopy-based results provided by the studies indicated the presence of functional groups hallmarking these compounds. These included N–H, C=O, C=C, COO^−^, N–O and C–N stretching. These compounds referred to as secondary metabolites had been shown previously to have biocidal activity against malaria parasites [83–91]. The mechanisms of action through which nanoparticles induce reduction in parasite growth rate and death are still clearly elusive. However, these could elicit their lethal action by operating on the genomic material of parasite, its surface membrane or even intracytoplasmic elements such as enzymes [35, 92]. The elucidation of mechanisms of antiplasmodial action is under intensive investigation. Karthik and colleagues reported the administration of gold NPs was associated with both a high TGF-β and low TNF production in mice infected with *P. berghei*; drawing the immunomodulatory role of metal NPs [61].

Finally, a few studies reported a toxic action of nanoparticles tested for their antiplasmodial potential. Indeed, a few of them reported nanoparticles had elicited toxic action against human cancer cell lines outlining thereby their possible but interesting anti-cancer potential. This is consistent with previous studies [93]. On the other hand, one study included in this review reported severe adverse effects and death cases caused by metal nanoparticles [61]. This brings back on the table the issue on the harmfulness of nanoparticles to humans. The question has been well documented [94–97], and implies that the evaluation of toxic potential of any new products is a crucial and composite step in the drug design. Thus, it would be important to include the evaluation of cytopathic effects of nanoparticles when their antimicrobial effect is evaluated.

### Future considerations

The some following researches worth addressing in future:

1. According to the 17 publications considered in the current review, most were conducted in India; and only one was conducted in Africa (South Africa). It is somewhat paradoxical as the African continent constitute the bulk of the total malaria burden and has an incredible diverse flora [3, 98]. Thus, there is need for more studies in this field in malaria endemic countries in this continent;
2. The mode of action through which metal nanoparticles elicit their biological effects is still elusive; thereby calling out to address this issue in future;
3. It is likely that many factors such as size and shape of NPs greatly influence their biological activities. Bioinformatics and modelling studies would be helpful to understand the real influence of these some abovementioned factors;
4. Seven out of seventeen papers included in the review addressed the toxic potency of metal NPs; of which three reported significant toxicity against non-target organisms [51, 61, 62]. This finding put in light conflictual results on this issue and point out a need for more extensive studies on NPs toxicity prior to any development of anti-malarial drug.
5. Finally, great discrepancies in methodological approaches were recorded in the 17 reviewed publications; from the process NPs synthesis to methods of evaluating their antiplasmodial activity. Thus, in order to compare the results of different studies, it would be interesting to standardize the methodology for evaluating the antiplasmodial activity of green nanoparticles.

## Limitations of the study

Articles written in English and French were included in the present review and as a result may result in selection bias.

## Conclusion

This review points out certain advantages in terms of rapidity and eco-friendliness of using living organisms such as plants for synthesis of metal nanoparticles. It provides a global overview on the antiplasmodial potential of these nanomaterials highlighting their usefulness as promising sources for new anti-malarial drugs. The review also highlights unanswered questions regarding the exact mechanism through which these NPs elicit their cytotoxic actions against the parasite, and the need for further studies addressing the issue. Lastly, the review underscores the need to conduct detail studies on the safety profiles of available nanoparticles prior to use in humans.

## Supplementary information

**Additional file 1.** PRISMA 2009 flow diagram.

**Additional file 2.** PRISMA 2009 checklist.

## Data Availability

All data generated or analysed during this study are included in this published article and its additional files.

## Abbreviations

AAS: atomic absorption spectroscopy
ACT: artemisinin-based combination therapy
AE: aqueous extract
AgNPs: silver nanoparticles
ART: artemisinin
AuNPs: gold nanoparticles
bw: body weight
CQ: chloroquine
DLS: dynamic light scattering
DRIFT: diffuse reflectance infrared Fourier transform
EDAX: energy dispersive X-ray
EDS: Electron Diffraction Spectrophotometer
EMBASE: Excerpta Medica Database
FESEM: field emission scanning electron microscopy
FTIR: Fourier-transform infrared
GMC: global malaria control
GMS: Greater Mekong subregion
GTS: Global Technical Strategy
HF: health facility
HIV/AIDS: human immunodeficiency virus/acquired immunodeficiency syndrome
HR-TEM: high resolution transmission electron microscopy
IC_50_: 50% inhibitory concentration
ICDD: International Centre for Diffraction Data
IPTp: intermittent preventive therapy in pregnancy
IRS: indoor residual spraying
JCPS: Joint Committee on Powder Diffraction Standards
LLINs: long-lasting insecticides-treated nets
MeSH: Medical Subject Headings
MHC_10_: minimum haemolytic concentration resulting in 10% haemolysis
MTT: 3-(4,5-dimethylthiazol-2-yl)-2,5-diphenyltetrazolium bromide
NC: negative control
PBMCs: peripheral mononuclear cells
PC: positive control
PRISMA: Preferred Reporting Items for Systematic Reviews and Meta-Analyses
RBC: red blood cells
SAED: size and elected area diffraction
SEM: scanning electron microscopy
TEM: transmission electron microscopy
TFG: tumour growth factor
TNF: tumour necrosis factor
NPs: nanoparticles
WHO: World Health Organization
