# Supplementary material for: A systematic review on anti-malarial drug discovery and antiplasmodial potential of green synthesis mediated metal nanoparticles: overview, challenges and future perspectives
Source: Malar J. 2019 Oct 3;18:337. doi: 10.1186/s12936-019-2974-9 (PMC6775654; doi:10.1186/s12936-019-2974-9)
Supplement: Supplementary file 1 — Additional file 1. PRISMA 2009 flow diagram. [file 12936_2019_2974_MOESM1_ESM.doc]

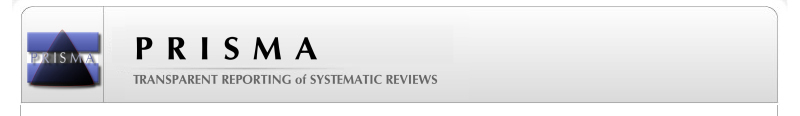
**PRISMA 2009 Flow Diagram**

**Screening**

**Included**

**Eligibility**

**Identification**

**Records identified through database searching**
(n = )

**Additional records identified through hand searching, Google and Google scholar**
(n = )

**Records after duplicates removed**
(n = )

**Records screened**
(n = )

**Records excluded**

**Full-text articles assessed for eligibility**
(n = )

**Article excluded**

**Studies included in qualitative synthesis**
(n = )
